# IMAGINING QUEER FUTURES BEYOND BOUNDARIES: A NARRATIVE ANALYSIS OF CREATIVE WRITING

**DOI:** 10.1093/geroni/igac059.540

**Published:** 2022-12-20

**Authors:** Sarah Jen, Rebecca Jones

**Affiliations:** University of Kansas, Lawrence, Kansas, United States; Open University, Milton Keynes, England, United Kingdom

## Abstract

Scholars have called for “queering aging futures” beyond normative assumptions or scripts (Sandberg & Marshall, 2019), which is well-aligned with queer theory’s Cruising Utopia which suggests “cruising ahead” toward a queer utopian future that is not yet possible (Muñoz, 2009). Due to emphasis on form rather than content, narrative analyses enable the reimagining of queer futures not bound by material realities. This study presents a narrative analysis of 40 pieces of creative writing in Bi Women Quarterly (BWQ) that examine aging. Authors used writing to queer stories of relationships, activism, and aging. Many used incoherent, non-linear, and dreamlike or omnipotent storytelling to queer narratives, allowing them to “cruise” across time and versions of themselves, imagining futures that were new and unscripted. Narrative analysis allowed researchers to examine choices authors made in taking agency through storytelling. Findings indicate that queer people are well positioned to queer expectations of successful old age.

